# Delays and Discrepancies in the Care of Older Injured Patients Across the United Kingdom: A Cohort Study

**DOI:** 10.7759/cureus.77689

**Published:** 2025-01-19

**Authors:** Timothy M Morris, Tom W Walshaw, Emily Winn, Thomas E Baldock, William G Eardley

**Affiliations:** 1 Trauma and Orthopaedics, South Tees Hospitals NHS Foundation Trust, Middlesbrough, GBR; 2 Trauma and Orthopaedics, Newcastle University, Newcastle upon Tyne, GBR

**Keywords:** cancellation rate, fragility proximal femur fracture, geriatric, surgical delay, trauma

## Abstract

Introduction

The average age of patients undergoing orthopaedic trauma surgery increases with over half of these procedures performed on older patients (over 60 years old). Despite this, the treatment of older trauma patients varies widely from their younger counterparts. Whilst diverging demographics and differing management are previously reported, no data is available regarding injury characteristics, comparative delays, cancellations or escalation to other lists. Through an analysis of the ORTHOPOD database, we provide a unique insight to improve understanding and challenge the current status of anecdotes and dogma.

Methods

A total of 22,585 patients from 83 hospitals (61 trauma units (TUs) and 22 major trauma centres (MTCs)) across the United Kingdom (UK) admitted over two months (August 22, 2022, to October 16, 2022) were analyzed to determine contemporary care in older patients within the current pathways.

Results

Older adults dominate the trauma caseload with 66% of operations being performed on this cohort and fragility proximal femur fracture (FPFF) patients alone accounting for over 1,500 more operations than for all fractures in younger adults (6369 vs 4806). Older trauma patients wait 40% and 60% longer for surgery as inpatients and outpatients, respectively. They also experience 25% more cancellations and are more likely to be managed entirely within a TU, be treated as an inpatient and sustain lower limb fractures. Conversely, they are one-third less likely to be escalated to elective lists. Regarding FPFF patients specifically, 70% are treated in TUs but, if treated in an MTC, both the cancellation rate and inpatient surgical delay are 25% higher than in TUs.

Conclusion

This analysis demonstrates the inequity in treatment received by older trauma patients. Despite comprising most of the UK’s trauma burden, this cohort waits significantly longer for surgery as both inpatients and outpatients and experience higher cancellation rates. This is despite these patients being frailer and more likely to be treated as inpatients since their injuries are often severe, for example, all femoral fractures are more common in older adults. Furthermore, they are less likely to be escalated to elective lists. This corroborates the need for initiatives to integrate dedicated patient pathways for older injured patients within trauma networks, for example, older injured adult trauma multi-disciplinary teams.

## Introduction

The proportion of the population that is older has continued to increase over recent decades. In 2021, those over 60 years of age comprised one-fifth of the population and accounted for two-thirds of population growth [1,2]. Resultantly, older females injured by low-energy transfer mechanisms dominate orthopaedic emergency admissions [3-5]. Simple falls account for over 80% of trauma cases. Half of fractures in men and 80% of fractures in women occur in those aged 70 or older [6]. In the United Kingdom (UK), the average age of those most severely injured (major trauma) has increased by almost 30 years within 30 years, from 36.1 to 63.6 years old between 1990 and 2020, respectively [3,7]. Additionally, almost half of all trauma operations are performed on patients over 60 years old, the largest cohort requiring surgery after injury [8]. This is not unique to the UK, with the percentage of older trauma patients more than doubling in Germany between 1993 and 2013 (from 16.5% to 37.5%, respectively) [9]. Despite low-energy transfer mechanisms, the injuries sustained by older patients are nonetheless severe [5,10].

This demographic change compromises healthcare systems, as it has outpaced system evolution. The current system in the UK is based on Regional Trauma Networks which primarily comprises two types of hospitals: major trauma centres (MTC) and trauma units (TU). In this system, those patients with "an injury or combination of injuries that are life-threatening and could be life-changing because it may result in long-term disability" should bypass their nearest, non-specialist hospital (TU) and instead be transferred to the MTC [11]. The MTC additionally treats less complex patients from their locality who are admitted directly to the hospital. Although, in this system, it would be envisaged that MTCs would treat most major trauma patients, this is inaccurate. Dixon et al. reported that most UK major trauma patients present to non-specialist hospitals and over 80% of trauma patients are treated solely by the hospital that they present to [4]. Double the number of seriously injured older patients are treated solely in a non-specialist trauma unit (TU) on a non-cohorted orthopaedic ward than in an MTC, with initial care in the emergency department being provided predominantly by junior clinicians [3]. Indeed, over 70% of trauma patients, with an injury severity score (ISS) of more than eight, presented to a TU and less than 10% were transferred to a MTC despite the injury severity being comparable [4]. The reducing frequency of transfers to MTCs was found to begin in patients as young as 50 years old with a further drop at 70 years old [12]. We postulate that this system may result in severely injured older trauma patients being concentrated within under-resourced, non-specialist units and receiving sub-standard care, as reflected by surrogate markers such as greater surgical delays. This contrasts starkly with paediatric trauma patients since, despite paediatric trauma patients comprising less than 5% of the trauma population, the majority are transferred directly to a specialised paediatric MTC [4,13]. The reasons for this appear complex, however, diagnostic nihilism and under-triage of the older trauma patient, resulting from a lack of "elderly-specific" triage criteria, are widely cited within the literature [5,14,15]. Put simply, older trauma patients make up the bulk of the significantly injured in the UK but are managed through a pathway that younger patients with fractures of the same bone are not.

This study aims to explore this phenomenon from the perspective of the entirety of the trauma population. We have assessed all patients being operated on in the 90 UK hospitals included in this study to reduce the skew of the evidence pertaining to those with major trauma only in order to identify the perioperative management of all older patients. We do this through analysis of the ORthopaedic Trauma Hospital Outcomes - Patient Operative Delays (ORTHOPOD) database.

It should be noted that this work has previously been presented as a poster at the Royal College of Emergency Medicine Annual Scientific Conference on September 26-28, 2023, and as a podium presentation at the British Orthopaedic Trainee Association (BOTA) Academic Congress on December 1, 2023, and was posted to the Social Science Research Network (SSRN) preprint server on May 6, 2024.

## Materials and methods

The method involved in the ORTHOPOD study has been previously reported by Baldock et al., 2023 [8].

Inclusion criteria

All adult trauma patients with bony injuries (excluding in the hand, foot or spine) were admitted to participating hospitals from August 8 to October 16, 2022, and were operated on by October 31, 2022.

Exclusion criteria

Pediatric patients, bony injuries affecting the hand, foot, or spine, and soft tissue injuries were excluded.

Three hospitals initially completed a one-month pilot study (July 18 to August 18, 2022). This resulted in the refinement of data fields and an alternative identifier was sought after the patient’s NHS number was determined to be unusable as a consequence of recent information governance (IG) changes. Collaboration from hospitals across the UK was promoted via social media utilising the platforms of the British Orthopaedic Association (BOA), BOTA and Orthopaedic Trauma Society (OTS).

This study had two independent arms:

Arm 1: caseload and theatre capacity

Data was captured prospectively each Monday morning at 08:00 throughout the period of data collection. The data was obtained to identify the number of theatre lists available at each site. Day case/ambulatory lists were defined as those lists on which patients were solely treated as outpatients, i.e., admitted and discharged to and from the patient’s usual residence on the same date of surgery.

Arm 2: patient, injury, and management demographics

The time of diagnosis was characterised by the timing of the initial confirmatory radiograph. The time of surgery was characterised by when the patient arrived in the anaesthetic room.

The Health Research Authority’s decision tool clarified that neither research nor ethical approval was necessary. Local approval was obtained from the relevant service evaluation teams; this along with the REDCap (Research Electronic Data Capture) registration and information sharing agreement were returned to the lead site. IG approval was granted by the lead site - James Cook University Hospital (JCUH) and "Data Access Groups" were created based on the hospital’s Information Sharing Agreement.

Two quality checks were carried out during the study, at the middle and end of the study (October 14-18 and November 5-8), to rectify absent or anomalous data entries. Local leads reviewed their site’s individual spreadsheet whilst the primary study team reviewed the collective data. This led to the exclusion of a single hospital since they could not collect data contemporaneously.

Data management and analysis

A secure, web-based platform named REDCap (Vanderbilt University, Nashville, USA) was utilised for data collection. This was then stored on servers with JCUH. As this study is based on a subset of the main dataset, we further condensed the data using Microsoft Excel (Microsoft® Corp., Redmond, WA, USA). Subsequent division of data into younger and older adult populations was necessary to analyse these distinct cohorts. Data is presented as absolute numbers and percentages; continuous data is quantified as medians with interquartile ranges (IQRs). Two-tailed T-tests were performed to determine the statistical significance of our results, and a p-value of 0.05 or less was deemed statistically significant.

## Results

A total of 14,097 patients had operations on orthopaedic trauma lists between August 22, 2022, and October 31, 2022. Regarding treatment pathway, 10,911 patients were treated as inpatients and 3181 were treated as outpatients. The proportion of patients aged 60 years or older was 65.90%, of these 6369 (68.55%) were fragility proximal femur fractures (FPFF).

**Figure 1:**
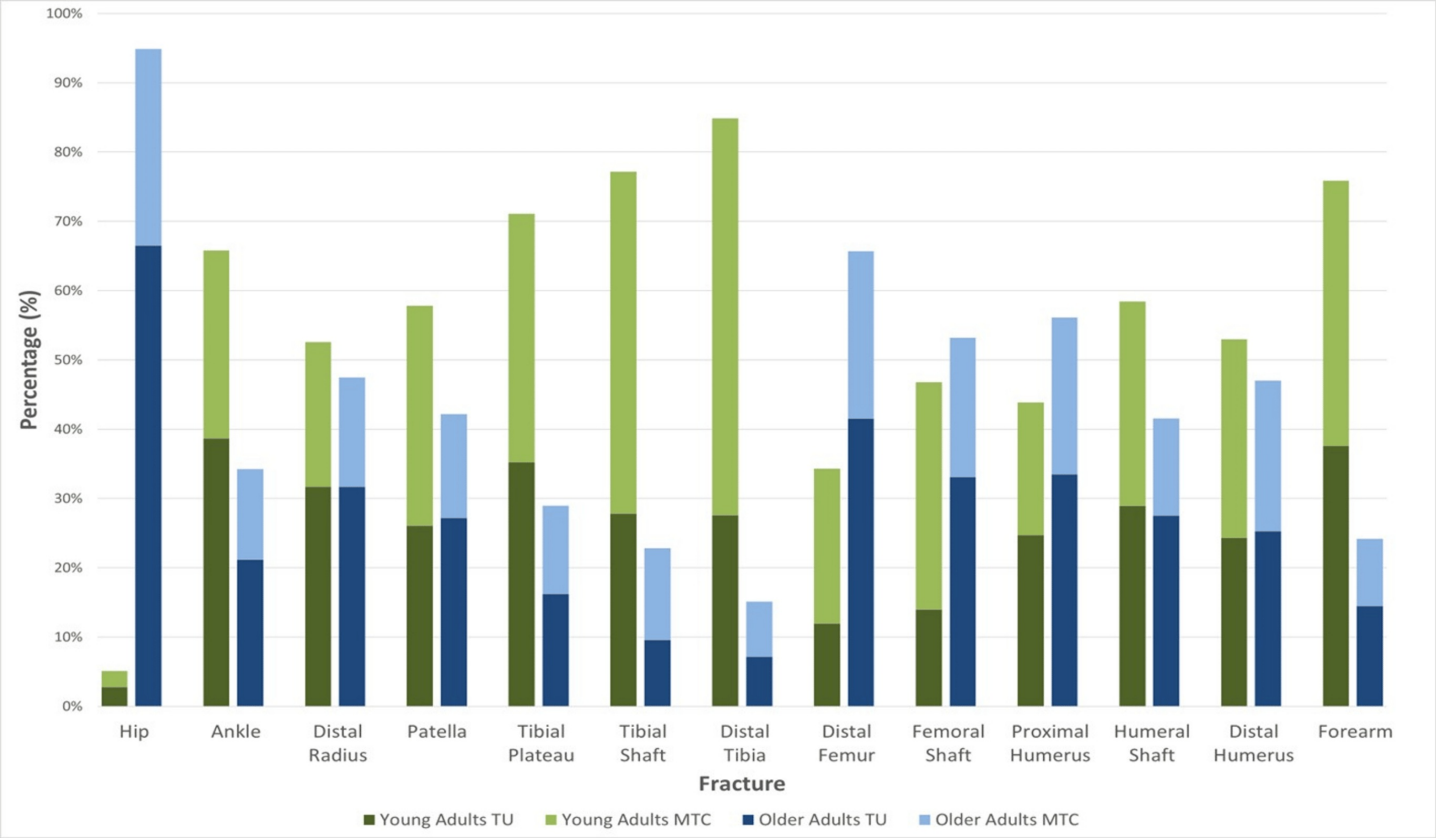
Proportions of Fractures in Both Young and Old Patients Across the UK A stacked bar chart demonstrating the spread of younger and older patients by both fracture type and environment. The darker and lighter colours represent TU and MTC, respectively. As with all graphs, green and blue represent younger and older adults, respectively. TU: Trauma Unit; MTC: Major Trauma Centre

Seventy per cent (9/13) of operatively treated fracture types are more common in younger adults (Figure 1). Indeed, almost half of these (4/9) are more than twice as commonly treated operatively in younger adults. Eighty per cent of upper limb fractures are more commonly operated on in younger adults, only the proximal humerus is more common in older adults. Similarly, over 60% (5/8) of lower limb fractures are more commonly fixed in younger adults. Nevertheless, hip fractures demonstrate an overwhelming preponderance by age, with older adults sustaining hip fractures more than 18 times more commonly.

**Figure 2:**
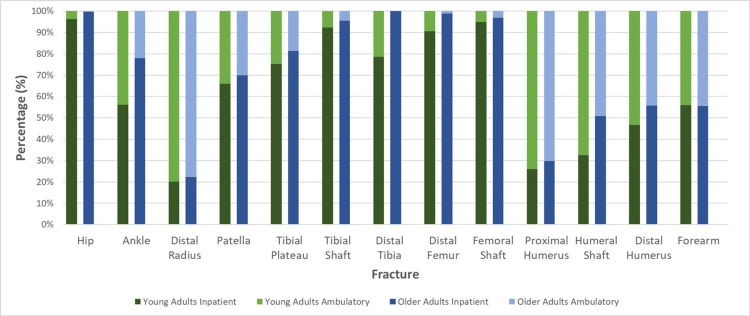
Inpatient vs Outpatient Treatment by Fracture Type A stacked bar chart demonstrating the treatment of fracture types for inpatient vs. outpatient. Each column represents the overall distribution for a certain cohort and is uniform across the fractures. The darker and lighter colours represent inpatients and outpatients, respectively.

On average, operatively treated orthopaedic patients are likely to be treated as inpatients, regardless of age (Figure 2). Indeed, 72.6% of our cohort were treated as inpatients. Older adults are 15.5% more likely to be treated as inpatients (p < 0.001). In 92% (12/13) of fracture types, a higher proportion of older adults were treated as inpatients than younger adults (p < 0.001); only in forearm fractures were younger adults 0.44% more likely to be treated as an inpatient (Figure 2).

Most fracture types (77%) demonstrated a statistically significant preference for either inpatient or outpatient treatment; only ankle, humeral shaft and distal humerus fractures failed to do so. Lower limb fractures are predominantly treated as inpatients regardless of age (p < 0.001). Indeed, every lower limb fracture was more commonly treated as an inpatient than any upper limb fracture. Similarly, distal tibial fractures were solely treated as inpatients in older adults whereas 21.5% of younger adults were treated as outpatients. Four lower limb fracture types (hip, tibial shaft, femoral shaft and distal femur) are treated as inpatients in more than 90% of patients, regardless of age (Figure 2). When these fracture types were identified as ambulatory, it is likely due to erroneous entries (e.g., errors in coding or incorporating non-unions into the data set) or extenuating circumstances not captured by the qualitative nature of this study.

Upper limb fractures were more commonly treated as an outpatient. Around 67.5% of younger upper limb fracture patients were treated as an outpatient (p = 0.019) and 80% of upper limb fracture types were more commonly treated as outpatients overall. Nevertheless, in older adults, only two upper limb fracture types were more commonly treated as outpatients (distal radius and proximal humerus).

As evidenced in Figure 2, the distal radius was the fracture most commonly treated as an outpatient with approximately 78.7% of patients being brought in for surgery after spending a period of time at home (p < 0.001).

**Figure 3:**
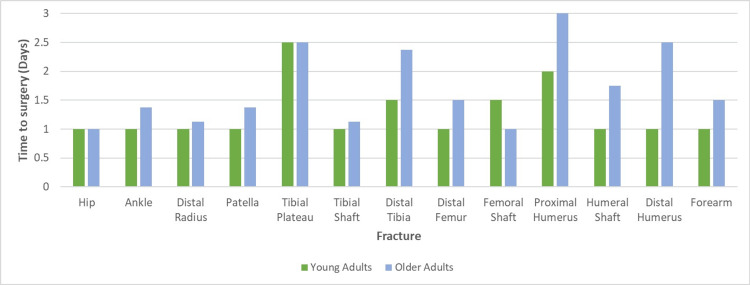
Median Time to Surgery for Inpatients

Seventy-seven per cent (10/13) of inpatient fractures wait less than 1.5 days for surgery, on average (Figure 3). Similarly, in 77% of fractures, older adult inpatients waited longer for surgery and in half of these, the wait was at least 50% longer. When a comparison was made for each fracture type, older inpatients waited, on average, almost 40% longer for surgery (p = 0.025). Overall, the median time to surgery for a young adult inpatient is one day, for an older adult, the average is 1.5 days.

Upper limb fractures waited 40% (or 12 hours) longer than lower limb fractures and proximal humerus fractures experienced the longest inpatient wait for surgery (three days). Older adult upper limb fractures wait longer than any other cohort for surgery, both as inpatients and ambulatory patients (Figure 3). Older adults with upper limb fractures wait 75% longer than their younger counterparts whilst older adults with lower limb fractures wait 37.5% longer.

**Figure 4:**
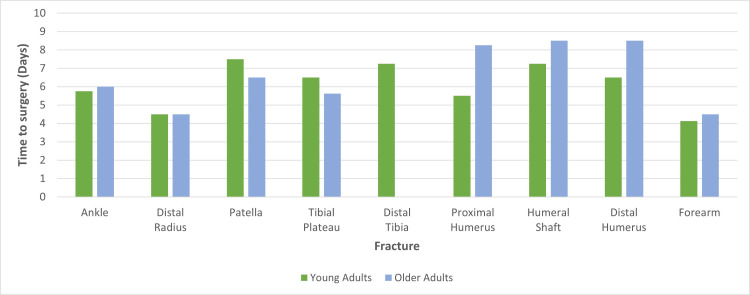
Median Time to Surgery for Outpatients (Excluding Fractures Treated Almost Exclusively As Inpatients)

Fifty-four per cent (7/13) of fracture types waited longer in the older patients and, when a comparison was made for each fracture type, an older outpatient waited approximately 58% longer for surgery (Figure 4). The average time to surgery was approximately five days longer when treated as an outpatient in both younger and older adults. Seventy-seven per cent of fractures treated as an outpatient waited one week or less and no fractures waited longer than eight days, on average. Distal radius and forearm fractures were the only fractures treated within five days on average (Figure 4).

Overall, upper limb fractures treated as outpatients waited approximately 12% (or almost 18 hours) longer than lower limb fractures. Additionally, ambulatory older adults with upper limb fractures waited almost three days longer for surgery than their younger counterparts (Figure 4).

Only 498 patients (3.53%) were escalated onto elective lists. Escalation onto an elective list refers to when trauma patients are placed on elective lists, which is often due to trauma caseload exceeding trauma operating capacity; ideally, these "escalated" patients would be lower-risk, healthier patients. Whilst older patients accounted for 60% of escalations onto elective lists, there were almost twice as many elderly patients in our cohort. Proximal humerus fractures were the most common fracture to be escalated to elective lists (10.9%). Moreover, upper limb fractures were 28% more likely to be escalated to elective lists and the four most commonly escalated fractures were upper limb. The likelihood of being escalated to an elective list was 30% higher for younger adults (4.2% vs 3.2%).

**Figure 5:**
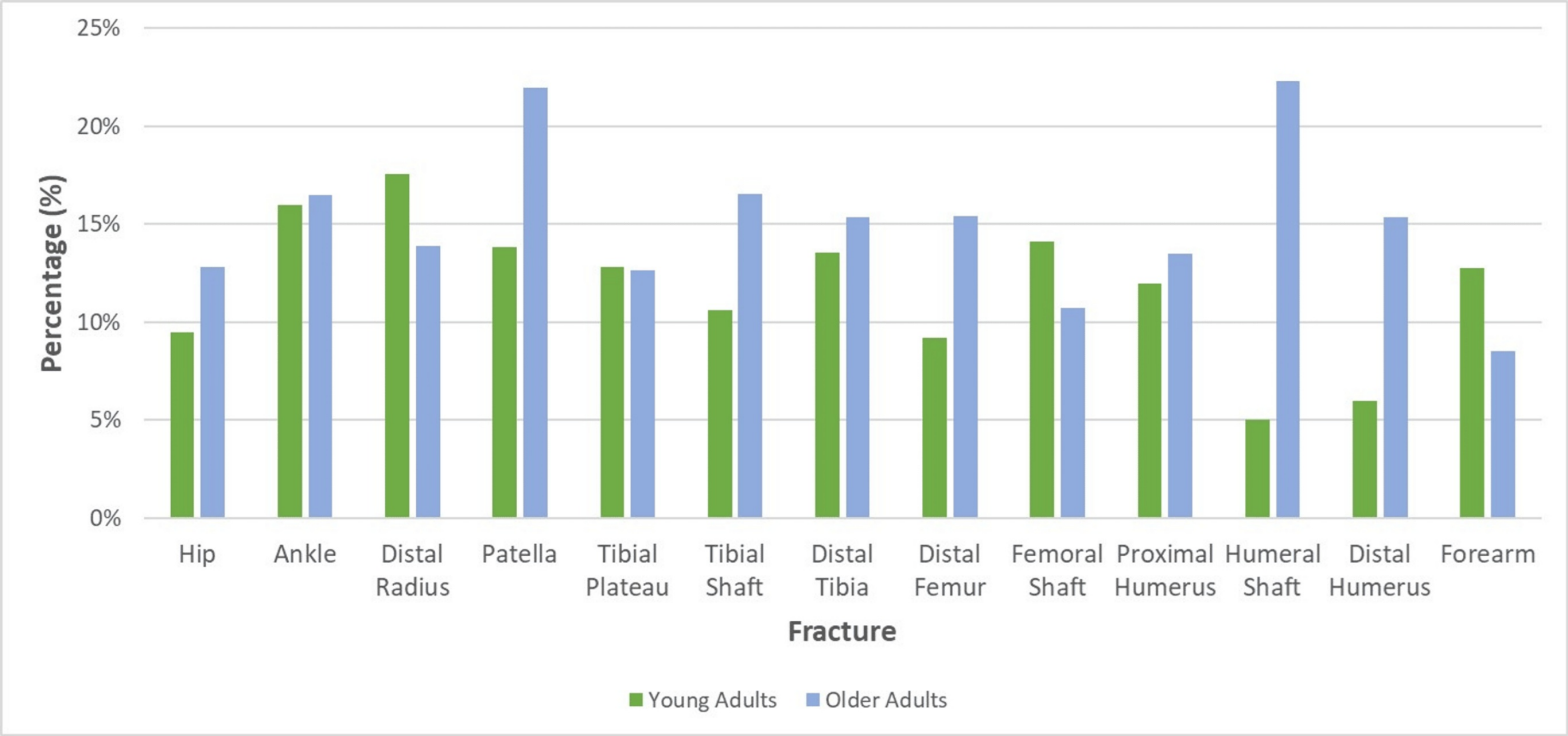
Mean Cancellation Rates, by Age and Fracture Type

The overall mean cancellation rate was 13.4%. Patella fractures were cancelled most at 17.91% whereas forearm fractures were cancelled least at 10.65% (Figure 5).

Older adults are 25% more likely to be cancelled than younger adults (p = 0.03), being cancelled more commonly in nine of the 13 fracture types (Figure 5). Overall, an average of 11.7% of younger adults and 15.7% of older adults were cancelled. Older adult upper limb fractures were cancelled 38% more commonly (p = 0.15) whilst lower limb fractures were cancelled 22% more commonly (p = 0.12). The fracture with the largest discrepancy in cancellations between younger and older adults, by percentage, was humeral shaft fractures with older adults being almost 4.5 times more likely to be cancelled (Figure 5). Indeed, older adults with humeral shaft fractures were the cohort that was cancelled most, by percentage, whilst younger adults with humeral shaft fractures were cancelled the least. Conversely, tibial plateau fractures had almost identical cancellation rates for both younger and older adults (Figure 5).

## Discussion

Older patients comprise the majority of cases undergoing surgery on trauma lists across the UK. These older patients accounted for two-thirds of patients in the study. Additionally, 85% of fracture types in older adults are more commonly treated by TUs. This corroborates previous findings that the UK’s trauma population is dominated by older trauma patient who presents to a TU, indicating that the infrastructure within our healthcare system needs to adapt to reflect this [4]. Whilst the emergence of MTCs significantly reduced morbidity and mortality rates in both the young, complex, polytrauma patient and paediatric trauma patient, this was not the case for older trauma patients [4]. Indeed, in a single-centre study, by Barr et al., the care of older trauma patients suffered directly from the hospital becoming an MTC with higher rates of post-operative complications, longer delays to surgery and increased 30-day mortality rates [16]. This finding is unsurprising since the trauma burden in an MTC is less predictable with more complex patients often requiring extended operative times. Similarly, case turnover is high so list priority changes frequently in MTCs which often results in more cancellations and longer time to surgery as demonstrated by this study since MTC patients experienced approximately 16% more cancellations and waited 2.3 times longer than TUs. Additionally, Dixon’s review of the TARN (The Trauma Audit and Research Network) database revealed that this cohort’s initial care is not senior-led, with 60% of older patients not receiving consultant review in ED and then, when they are admitted, they are unlikely to be admitted to a major trauma ward [4].

Our study suggests that older patients may be experiencing inequalities in care. Despite their advanced age and frailer physiological state, in comparison to their younger counterparts, older patients wait 40% longer for surgery as inpatients and 60% longer as outpatients. As an inpatient, 77% of fracture types wait longer to be treated in the older adult and, in half of these, the wait is more than 50% longer. An explanation for this discovery could be that this frailer cohort also experienced 25% more cancellations. This issue is more prevalent in MTCs with older adults being cancelled 25% more commonly in MTCs than in TUs. These findings are concerning since some of the most serious injuries, e.g., all femoral fractures, are more common in this cohort in particular, yet they wait longer when compared against their peers treated in TUs, e.g., inpatient FPFF wait 25% longer in MTCs. Older adults with upper limb fractures had the longest time to surgery on average and this same cohort also experienced 38% more cancellations than their younger counterparts. This finding was mirrored to a lesser extent in lower limb injuries with the older patient experiencing 22% more cancellations than the younger adult. Finally, older adults were 30% less likely to be escalated to elective lists, which also contributes to their surgical delays and higher cancellation rates. Whilst this may potentially be due to older patients being less suitable for escalation, given their higher surgical and anaesthetic risk, escalation to other lists reduces surgical delay since elective cases are cancelled to accommodate trauma. This escalation only occurs when trauma load outweighs capacity since this has financial implications since elective operations carry a higher tariff [17].

The large number of cancellations observed in this study is unsurprising given recent evidence that has determined that cancellation rates within both UK and international orthopaedic departments have increased by over 40% in the last decade [18,19]. The reasons for this are complex and were outside the scope of this study; however non-clinical, "systemic failures", e.g., excess bed occupancy, are commonly implicated [20,21]. Wong et al. concluded that the need for a post-operative critical care bed and the presence of an emergency department within a hospital are independent risk factors for cancellations; this could explain the excess surgical delay observed in our MTC population [20]. In addition, other contributing factors include organisational inefficiency, clinical prioritisation and patient factors [20]. Patient factors are particularly important in elderly trauma patients since over 75% of this population has an American Society of Anesthesiologists (ASA) grade of 3 or above; therefore, they often require pre-operative testing and medical optimisation [19,22,23]. Perhaps, the most prevalent reason for surgical delay and excess mortality, in the elderly specifically, is anti-coagulant use since almost 40% of older injured adult patients and 60% over 75 years old are anti-coagulated [24].

Whilst sometimes clinically indicated, surgical cancellations reduce efficiency and increase costs. Similarly, they cause disruption to the patient and their families (with protracted functional and financial loss) and, most importantly, produce poorer outcomes [19,25-28]. These consequences have been well documented in FPFF patients, hence, the introduction of initiatives like the Best Practice Tariff (BPT). Surgical delays of as little as 24 hours have been shown to result in significantly increased length of stay and complications (including mortality) in orthopaedic patients as young as 55 years old [25-28]. In FPFF patients with a surgical delay of greater than 48 hours, increased mortality has been demonstrated at all time intervals, from in-hospital mortality to one-year mortality [26,27]. Perhaps, the most significant of these findings was made by Paul and Issac who demonstrated an eight-fold increase in in-hospital mortality in FPFF patients with a surgical delay greater than 48 hours thus highlighting the importance of timely intervention in this older, frail cohort [27].

What may therefore be necessary is a transformation of current pathways in the care of the older trauma patient with the advent of dedicated older injured adult trauma teams delivering specialised multi-disciplinary team (MDT) care within existing hospitals. Kojima et al. discovered that patients admitted to trauma centres treating the highest proportions of older injured adult trauma had the best outcomes with an estimated 40% reduction in one-year mortality, for example [14]. These findings are unsurprising given the nuances of care of older injured trauma patients since these patients possess altered physiology and high levels of polypharmacy, dependency and co-morbidity. Whilst older orthopaedic patients are often reviewed by geriatricians in the UK, a dedicated MDT working via a separate pathway to adult trauma patients would concentrate resources to deliver more focused care and, ultimately, improve outcomes. This pathway would culminate in dedicated theatres managing older injured adult trauma. Whilst this has not yet been attempted for trauma, evidence from dedicated FPFF lists is promising; cancellation rates have been halved with simultaneous reductions in the time to surgery and length of stay [21,29].

In addition to the inequalities experienced by older adults, we have also discovered other areas for improvement within the UK’s trauma care. Whilst over 70% of operatively treated trauma patients receive inpatient care, ambulatory patients wait approximately five days longer for surgery. Although this may be due to these patients likely sustaining less severe injuries, this delay is significant since a trend has emerged within the literature that outpatient procedures typically experience significantly higher rates of cancellation than inpatient procedures, this was not assessed within our study, however [18,19]. Similarly, upper limb fractures wait significantly longer than lower limb fractures as both inpatients and outpatients, this is significant because upper limb fractures are far more likely to be treated as outpatients that, as a cohort, already wait longer for surgery. This extended time to surgery in upper limb fractures may be due to the need for specialist input which may be reflected in escalation to an elective list being more common in these fractures.

Limitations

Whilst we have provided a comprehensive and contemporaneous assessment of orthopaedic trauma care within the UK, as with any study, limitations exist. Since this study was based on data collection from numerous collaborators, correct data input is paramount. Significant attempts were made to limit inaccuracies, with multiple checks throughout the data collection period, however, no study of this magnitude can be errorless. Nevertheless, we believe that any errors that have been included do not alter the overall findings meaningfully.

Other, perhaps more significant limitations, are that we did not collect co-morbidities or the functional status of patients and we similarly did not collect patient outcomes. Whilst medium- to long-term follow-up would have been problematic, short-term follow-up would have been useful to assess the utility of ambulatory pathways amongst the older patients since these patients have a higher risk of overnight admission/readmission following day-case procedures. Finally, our data did not document if ambulatory patients underwent procedures on dedicated day-case theatre lists, we collected which patients were suitable for ambulatory pathways and who were operated on as ambulatory patients but not if their procedure was on a dedicated day-case list.

## Conclusions

For the first time, the distribution and discrepancies in the management of numerous fracture types within clearly defined populations have been identified. This analysis has demonstrated the significant differences in treatment received by older trauma patients. This cohort, which comprises most of the UK’s trauma load, waits significantly longer for surgery as both inpatients and outpatients and experience much higher cancellation rates despite their advanced age and frailer physiological state. Furthermore, older adults are less likely to be escalated to elective lists. All of this corroborates the need for initiatives to prioritise the care of older injured trauma patients, e.g., dedicated MDTs.
